# Revealing disparities in representation in knowledge generation and guideline development

**DOI:** 10.1186/s12913-024-11958-1

**Published:** 2024-11-30

**Authors:** Carlos P. B. Almeida, Afom T. Andom, Alain Casseus, Jacquelyn-My Do, Alain Gelin, Leonid Lecca, Maxo Luma, Michael Mazzi, Carole D. Mitnick, Jean Claude Mugunga, Melino Ndayizigiye, Natalie Nguyen, Meseret Tamirat, Girum Tefera, Sterman Toussaint, Marco Tovar, Christine Tzelios

**Affiliations:** 1 Universidade Federal Do Sul E Sudeste Do Pará, Marabá, Brazil; 2 Harvard Medical School, Boston, USA; 3 Partners In Health Lesotho, Maseru, Lesotho; 4 Partners In Health, Boston, USA; 5 Zanmi Lasante, Croix-des-Bouquets, Haiti; 6 Partners In Health Liberia, Monrovia, Liberia; 7 Partners In Health Sierra Leone, Freetown, Sierra Leone; 8 Cornell University, Ithaca, USA; 9 Insitituto de Investigación Nutriciona, Lima, Peru; 10 Universidad Peruana Cayetano Heredia, Lima, Peru; 11 Comite de Expertos, Red Peruana de Tuberculosis Pediátrica, Dirección de Prevención y Control de la Tuberculosis, Ministerio de Salud, Lima, Peru

**Keywords:** Multidrug-resistant tuberculosis, Guideline development, Global health disparities, Decolonization, Epistemic injustice

## Abstract

**Background:**

Multidrug-resistant tuberculosis (MDR/RR-TB) is a major global health challenge, disproportionately affecting low- and lower-middle-income countries (LLMICs). The World Health Organization (WHO) generates guidance to address the problem. Here, we explore the extent to which guidance and related knowledge are generated by experts living in the most-affected countries and consider the results in the context of the movement to decolonize global health.

**Methods:**

We examined the composition of World Health Organization (WHO) MDR/RR-TB treatment Guideline Development Groups (GDGs) from 2016 to 2022. We classified GDG members according to the MDR/RR-TB burden and World Bank income level of the country of their institutional affiliation. We also searched PubMed to identify peer-reviewed publications from 2016 to 2023 which emanated from individual-patient-data meta-analysis like those done for Guideline review, and classified the publication authors according to the same indicators.

**Results:**

There were 33 high-burden MDR/RR-TB countries during the time period. Of these, 72.1% were LLMICs and none was high-income. In contrast, only 30.3% of WHO GDG members and 10.4% of peer-reviewed publication authors were from LLMICs. Representatives from high-MDR/RR-TB-burden countries comprised 34.3% of WHO GDG members and 14.7% of authors of guideline-related publications.

**Conclusions:**

The important imbalance between the geographical distribution of lived experience with MDR/RR-TB and the distribution of individuals generating knowledge and guidance on treatment of MDR/RR-TB can have clinical and resource implications. Countries may reject or defer guideline adoption because of a mismatch between that guidance and local disease epidemiology. Funding conditioned on compliance with guidelines can exacerbate health inequalities. The movement to decolonize global health considers representation disparities as epistemic injustice, that is unfair treatment in the process of generating, sharing, or receiving knowledge. Reform is possible in many of the institutions involved in generation of global health knowledge, such as: meaningful participation of LLMICs in projects as a requirement for research funding, improved attention to the epistemic and geographical location of journal editorial staff, and broader inclusion in guidelines committees. Better alignment of participation in knowledge generation with burden of disease holds potential for reducing inequality and improving relevance of guidance for the lived experience with MDR/RR-TB.

**Supplementary Information:**

The online version contains supplementary material available at 10.1186/s12913-024-11958-1.

## Background

Multidrug-resistant and rifampin-resistant tuberculosis (MDR/RR-TB) pose a significant global health challenge, particularly in low-income countries (LICs) and lower middle-income countries (LMICs), which bear the largest burden of MDR/RR-TB cases [1]. To respond to this threat, the World Health Organization (WHO) periodically issues guidance for the management of MDR/RR-TB; in the last six years, four sets of updated Guidelines have been released. The intended audience for these documents is national TB program (NTP) managers, advisors, clinicians, donors, and other stakeholders in countries with MDR/RR-TB. Peer-reviewed publications are also produced from the analyses that are performed to inform the WHO Guidelines [2–5]. These publications may reach a broader audience than the guidelines, with potential to influence research priorities and funding and reinforce the scientific legitimacy of the guidance. Uptake of recommendations included in guidance is decidedly inconsistent and may be influenced by limitations in funding, healthcare worker training, healthcare infrastructure, and national legislation or policies [6, 7]. Low certainty of evidence, as graded by guideline committee members and which leads to conditional recommendations, has also been found to delay uptake [8]. And, it is postulated that various forms of diversity (i.e., race and gender) may affect the content of consensus documents by influencing which topics are addressed and prioritized [9]. Another heretofore unexplored, possible factor is the composition of guidance committees, i.e., their degree of inclusiveness of representatives from countries experiencing the greatest burden of MDR/RR-TB and from LICs and LMICs.

To begin to investigate this, here we explore the inclusion of people from LICs and LMICs (LLMICs): 1) on committees that conducted reviews for the last four WHO Guidelines for the treatment of drug-resistant TB and 2) among co-authors of related peer-reviewed publications. Specifically, we compare to the proportion of LLMICs in the high-burden MDR/RR-TB country list the distributions of the institutional affiliations of: 1) members in WHO Guideline Development Groups (GDGs) and 2) the authors of related peer-reviewed publications. We then examine these findings in the historical context of global health as a colonial endeavor and explore solutions within the literature on decolonizing global health.

## Methods

### Data collection and classification

Using the WHO lists of high-burden MDR/RR-TB countries [10], we classified countries dichotomously as high-burden or not for each year in which a WHO Guideline on MDR/RR-TB was published: 2016, 2018, 2020, and 2022 [2–5]. We also classified the countries as low-income (LI), lower-middle-income (LMI), upper-middle-income (UMI), and high-income (HI) for each year from 2016–2023, according to the annually updated World Bank income classification lists [11]. For each of the four WHO MDR/RR-TB Guidelines, we extracted the list of all GDG members and country of their institutional affiliation (Supplement S1). We classified each member by the country in which their stated institution was based according to its: 1) presence or absence in the list of high-burden MDR/RR-TB countries and 2) World Bank income level.

### Identification of articles linked to WHO MDR/RR-TB guidelines and classification of authors

We searched PubMed for articles that were published between January 2016 and August 2023 on MDR/RR-TB individual-patient-data meta-analyses (of the type) conducted for guideline review. We searched for the terms “individual patient data” OR “meta-analysis” in title or abstract and “multidrug resistant tuberculosis” as a MeSH term. We imported the results into rayyan.ai for screening. Articles were included if they were individual patient data meta-analyses of treatment safety or effectiveness. Two authors (JD, CDM) screened all abstracts for inclusion criteria; if insufficient information was available from abstracts, full text review was performed. Articles were excluded for the following reasons: not systematic reviews, individual patient data (IPD) not used, publication was not about MDR/RR-TB treatment effectiveness or safety, or the wrong publication type (i.e., commentaries, non-research letters to the editor, and protocol or methods paper). Authors’ institutional affiliations were obtained from the PubMed extraction via the easyPubMed R package (v2.13; Fantini 2019) to determine the country(s) of the institutions. If the affiliation field was missing or had errors in the extraction, we manually reviewed the paper and recorded details. We classified each author of a peer-reviewed article as we did for the GDG members, by the country in which their institution was based according to: 1) inclusion in the list of high-burden MDR/RR-TB countries and 2) World Bank income level. Authors from institutions in Taiwan and the Special Administrative Region of Hong Kong were classified as high-income and not high-burden MDR/RR-TB. For authors with multiple institutional affiliations, we used as primary the institution in a country that was classified as LLMIC and/or high-burden MDR/RR-TB. For example, an author with both a United Kingdom institutional affiliation and an India institutional affiliation would be classified as having an India affiliation.

### Analysis

We calculated the proportion of GDG members and peer-reviewed publication authors from institutions based in high- and non-high-burden MDR/RR-TB countries and the distribution of the individuals’ institution locations across World Bank income levels. We calculated proportions for each Guideline. Each author of a peer-reviewed publication was counted once per publication. To evaluate relative representation, we compared each of these indicators to the percentage of high-burden MDR/RR-TB countries that are LLMIC.

Data was downloaded for the WHO list of high-burden countries for MDR/RR-TB and World Bank income classification in csv files. Author lists extracted from the PubMed search results were output into an Excel spreadsheet. Proportions were calculated in Microsoft Excel version 16.80.

## Results

**Fig. 1:**
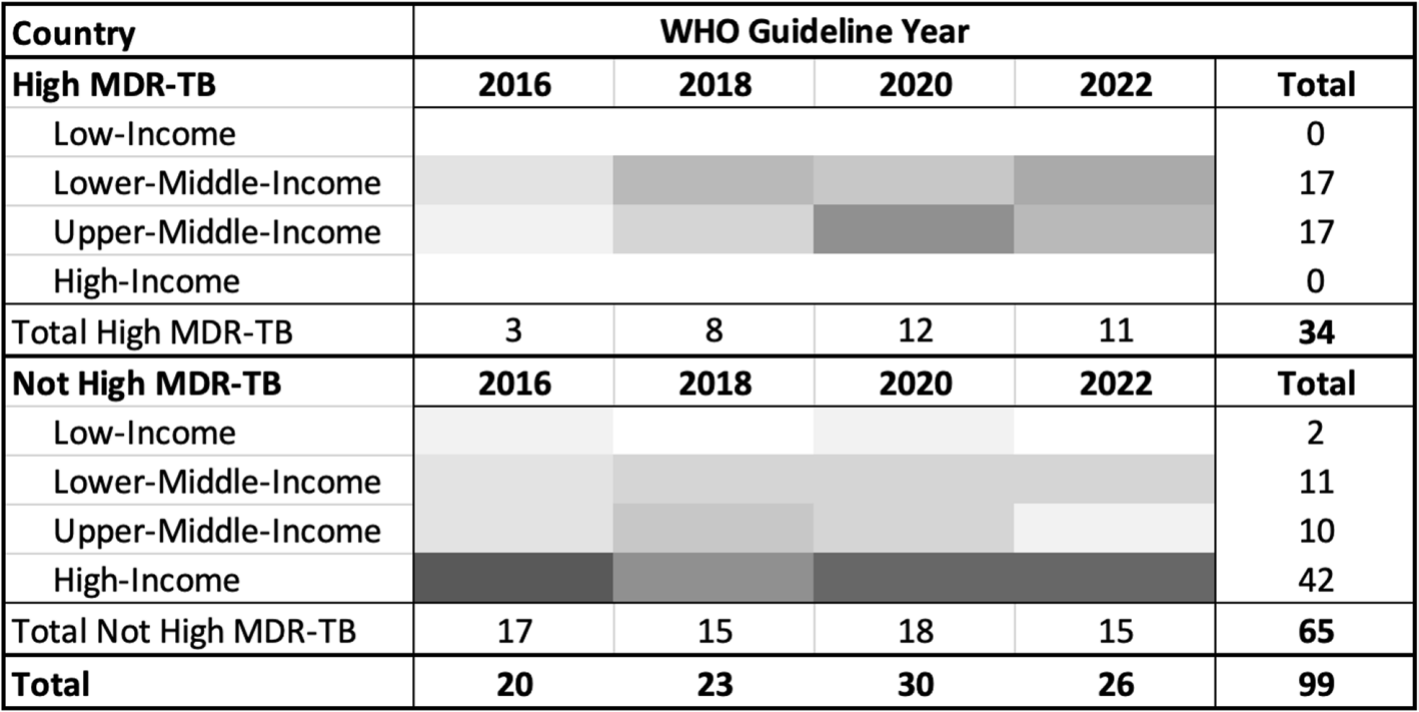
Representation of Countries in Guideline Development Group (GDG) and Corresponding Number of Committee Members (2016–2022). Legend: Shading represents the proportion of GDG members in the dichotomous MDR/RR-TB burden (gradient from light to dark indicates smaller to larger proportions) classification and World Bank income level, by WHO MDR/RR-TB Guideline publication year

Of the 33 countries designated as high-burden for MDR/RR-TB in the WHO lists for 2016–2020 or 2021–2025, 72.1% are classified as low or low-middle-income. This serves as the benchmark for evaluating representation. No high-income country is high MDR/RR-TB burden. In the four GDG committees, 99 members were affiliated with institutions located in 38 unique countries, with diversity increasing over time: 20 members came from 14 countries in 2016, 23 members came from 17 countries in 2018, 30 members represented 22 countries in 2020, and 26 members represented 20 countries in 2022. Only 34 of 99 (34.3%) GDG members were from MDR/RR-TB high burden countries (Fig. 1); 30.3% (30) were from LLMICs. 17.2% (17) were from high-burden LLMICs, far lower than the benchmark.

**Fig. 2:**
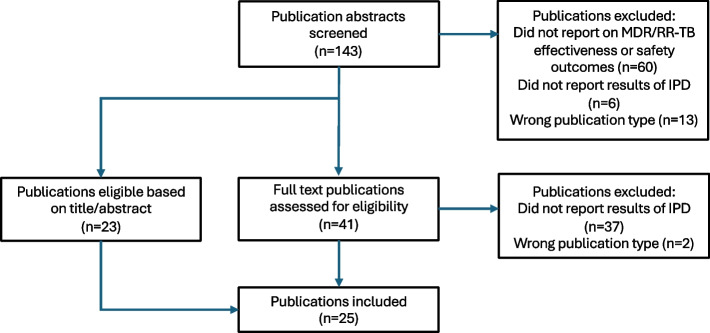
Article review and selection

**Fig. 3:**
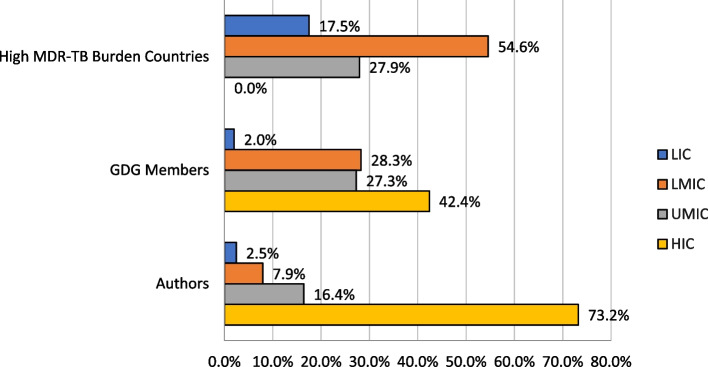
Distribution of high-burden MDR/RR-TB countries, membership composition of WHO MDR/RR-TB Guidelines Committees 2016–2022, and authorship of related publications stratified by World Bank Income Classification. Note (Fig. 3): GDG members and publication authors are categorized by country of their institutional affiliation

The PubMed search for articles published between January 2016 and August 2023 yielded 143 publications. During abstract screening, we excluded 79: 60 because they did not report on MDR/RR-TB effectiveness or safety outcomes, 6 because they did not report on an IPD, and 13 because they were the wrong publication type. An additional 39 were excluded during full text review: 37 because they were not IPD (see Fig. 2) and 2 because they were the wrong publication type. Ultimately, 25 articles were included for this analysis. There were 530 total authors (300 unique) across all publications. There was an average of 21.2 authors per publication (data not shown). Study authors were affiliated with institutions in 50 unique countries. Author institutions located in high-burden MDR/RR-TB countries accounted for 78 of 530 (14.7%) authors; 55 authors (10.4%) were from institutions in LLMIC countries. Only 34 authors (6.4%) were from institutions in countries that were both high-burden and LLMIC (Table 1). This is far lower than of the percentage of high-burden countries that are LLIMC (71.2%) (Fig. 3).

**Table 1:** Representation of non-unique authors in peer-reviewed publications of MDR/RR-TB individual patient meta-analyses published between 2016–2023, *N* = 530

Author Affiliation Country	N	%
**High MDR/RR-TB Burden**	**78**	**14.7**
Low-Income	0	0.0
Lower-Middle-Income	34	6.4
Upper-Middle-Income	44	8.3
High-Income	0	0.0
**Not High MDR/RR-TB Burden**	**452**	**85.3**
Low-Income	13	2.5
Lower-Middle-Income	8	1.5
Upper-Middle-Income	43	8.1
High-Income	388	73.2

## Discussion

Our analysis reveals an important imbalance between the geographic distribution of MDR/RR-TB, the disease for which knowledge is being generated for treatment guidance, and the geographic distribution of individuals involved in knowledge generation and interpretation. While the burden of disease is concentrated in LLMICs, and guidelines are written largely for LLMICs, knowledge generation is primarily left to individuals working in HICs that are low-burden MDR/RR. Specifically in this study, 73.2% of authors of peer-reviewed manuscripts and 42.4% of GDG members were affiliated with institutions in HICs while no high-burden MDR/RR-TB country falls into this World Bank income category.

These disparities are similar to those observed in publications on TB and other infectious diseases generally. In a systematic review of 1,593 TB, HIV, and malaria randomized controlled trials conducted in low- and middle-income countries from 1990 to 2013, 49.8% of first authors had low- and middle-income countries affiliations [12]. Similarly, a separate systematic review of 1,182 publications on infectious disease research conducted in Africa from 1980 to 2016 found that only 49.8% of first authors and 41.3% of last authors were affiliated with African institutions [13]. Several factors may explain these differences [12], including the concentration of funders and research institutions in HICs [14], potential biases in peer review [15], and the dominance of the English language in academic literature. Notably, most African-affiliated first and last authors were from Anglophone countries [13]. Gender inequality in global health authorship is also more pronounced in low- and middle-income countries [16], highlighting the range of characteristics on which disparities can be detected.

As noted, the disproportionate influence of HICs also carries over into representation among those using knowledge to establish policy. The WHO Handbook for Guideline Development states that GDG members should be identified through open calls for nominees [17] or from established technical networks and WHO Collaborating Centers. The majority of WHO Collaborating Centers for TB are currently situated in UMICs or HICs [18], a factor that may reinforce the imbalance. The WHO Handbook further specifies that GDG membership should “be balanced in terms of gender and geography” and include representatives from all regions where the guidelines will be used [17]. The composition of GDGs included in the present analysis likely satisfies these basic requirements. It is, however, important to consider some possible downstream effects of the observed underrepresentation of LLMICs in Guideline development.

While countries are sovereign entities that can choose their own policies around TB patient care, guidance can both influence and reflect funding available for TB. Normative guidance is intended to be relevant to all settings. Its applicability, however, can vary by the epidemiology of TB or the regulatory environment in a country. Rejection of the guidance—or adoption of innovation based on peer-reviewed literature before the change is codified in guidance—can compromise funding because some multilateral and bilateral funding for TB in high-burden countries (e.g., World Bank, Global Fund) is conditioned on compliance with WHO guidance [19]. Countries are then forced to choose either application of imperfectly matched guidance or external funding. Moreover the GRADE framework, used as the foundation for WHO and other normative guidance development, explicitly allows for consideration of “resource implications” in determining recommendation strength as part of the systematic approach to evaluating evidence and making recommendations [20]. This means that a recommendation can be classified as conditional at least in part because of the resources involved, which can in turn delay in-country regulatory processes. For example, the anti-TB drug, delamanid, which has remained expensive (approximately $1200/course in February 2024) [21] since its authorization by the European Medicines Authority in 2013, has been only conditionally recommended by WHO. This signals ambivalence, which has been shown to delay uptake [20]. In the case of delamanid, it likely contributes to the delayed inclusion of delamanid in the national guidance and the national formulary in some countries and to the continued inability of patients with TB to receive treatments containing this safe, oral anti-TB drug. Another approach taken when resources are considered is to alter guidance for LLMICs to favor lower-cost, inferior interventions over higher-cost, superior interventions [22]. This can perpetuate a double standard, whereby the countries in which the guidance developers work have a higher standard of care than in (some of) those for which the guidance is intended. Increased LLMIC representation in GDG might avert some of the uptake challenges that have been observed when guidance is generated by less diverse committees. This structure, and the double standard, effectively assume that current resource allocations and pricing structures are fixed and that no change is possible to broaden access to the highest evidence-based standard of care.

Another limitation of recent MDR/RR-TB guidance might reinforce the consequences of representation gaps. The GRADE framework allows for the inclusion of “values and preferences” in guidance development; however, patient-level data to inform these factors is often limited. For example, although various qualitative assessments of patient perspectives on treatment have been published, only two such papers – both unpublished – have been referenced across the four Guidelines: 1) in 2020, the results of 16 interviews with patients; 2) in 2022, a mixed-methods study describing patient perspectives on treatment [23]. Having more LLMIC representation could enhance insight on local values and preferences, with the caveat that even within countries, practitioner and patient values may differ importantly.

Many of the LLMIC—and some UMIC—high-MDR-burden countries are former colonies of present-day HICs. It is useful to consider the observed disparities in the context of the colonial origins of global health, their legacies, and contemporary attempts to decolonize the discipline. During the British colonial era, infectious diseases were considered a threat to commercial and military interests [24]. The demand for knowledge about these diseases led to the inception of microbiology as a scientific discipline and the establishment of the first tropical medicine research institutions in the UK, as well as the earliest international health agencies, which merged into the WHO after World War II [24, 25]. During the Cold War, power asymmetries in global health governance were exacerbated by neoliberal policies that limited LLMICs’ ability to expand healthcare and scientific infrastructure. For example, in the 1980s, Structural Adjustment Programs (SAP) conditioned loans from the International Monetary Fund and World Bank on adoption of strict austerity measures. These, in turn, eroded public health and social services spending [24]. In some areas, SAPs were even associated with worsened TB incidence and mortality [26]. Although twenty-first-century international agreements, such as the Paris Declaration on Aid Effectiveness (2005) and the Accra Agenda for Action (2008), have called for donor funding to align with local, rather than HIC, priorities, minimal progress has occurred [27].

In response, a movement has emerged to decolonize global health. It underscores that coordinated efforts across multiple sectors are required to redress prevailing inequities in global health collaborations. The ethics segment of the movement has provided a framework for understanding how unfair treatment in the process of generating, sharing, or receiving knowledge–known as epistemic injustice— [28] is perpetuated in global health. Key strategies to address epistemic injustice are described below, with application to the case of MDR/RR-TB publications and guidelines.

### Funder

Most TB research funding comes from bilateral and multilateral organizations in HICs [29] and is preferentially awarded to researchers within those countries [14]. HIC researchers therefore dictate priorities and resource allocation, with LLMIC collaborators often relegated to passive roles [14]. LLMIC-based co-authors of the present manuscript also identify insufficient compensation and/or protected time as obstacles to meaningful participation in research. Reform measures could include: 1) intentional solicitation of input from LLMIC researchers in TB-research priority-setting exercises for funding institutions. Public calls, for example by the US National Institutes of Health/National Institute of Allergy and Infectious Diseases (NIH/NIAID) on TB research priorities [30], may not be commonly answered by LLMIC researchers; direct (compensated) solicitation of input may be required. 2) directly funding LLMIC researchers and institutions at levels equivalent to those at which HIC researchers are funded. We note two examples where this is not the case. First, direct costs for the NIAID R01 mechanism for international investigators [31] are capped at 25% of the budget allowed for the general R01 mechanism. Second, NIH indirect costs are generally capped at 8% for foreign awardees while US institutions negotiate rates. These rates are variable, but in a convenience sample of public and private universities in the state of Massachusetts, on-campus research indirect rates exceed 50% at all except one in 2024 [32–36] 3) establishing policies that require equitable decision-making and authorship for continued funding [14, 37].

### Journal

Across major global health journals, only one-third of editors and editorial board members are from LLMICs [12] – an imbalance that positions HIC actors as the “gatekeepers” of knowledge dissemination. To address this, leading TB journals must increase editorial representation from the countries where the research they publish is conducted. As one example, PLoS Global Public Health, which aspires to be a “home for work about the Global South, by the Global South,” states that half of its Section Editors and Academic Editors are from the Global South [38]. Additionally, in recent years, a group of major research funders has required researchers to publish in exclusively open-access (OA) journals [39]. While broadening potential readership, this mandate could prohibit LLMIC researchers from publishing if OA fees are not reduced or sufficiently covered by research funding [40]. The International Journal of Tuberculosis and Lung Disease, for example, has opted for two journals, one subscription and one OA [40]. To illustrate a different approach, PLoS Global Public Health launched the Global Equity Model, whereby partner institutions pay a flat annual fee, reflective of their country’s World Bank income level, to enable their researchers to publish in the open-access journal without publication fees [41]. Other journals that publish TB research should consider mechanisms that facilitate LLMIC engagement in scholarly discourse through financially accessible readership and publication.

### Guideline development

Even when LLMICs are represented in GDGs and other decision-making bodies, chosen members are often those with connections to HIC powerholders and perceived credibility according to Global North standards [42]. Epistemic justice necessitates real diversity, which can be achieved by harnessing the heterogeneous experience and knowledge of community stakeholders, prioritizing those most affected by TB policies, including patients, caregivers, or healthcare workers [43, 44]. The WHO’s “Guidance on engagement of communities and civil society to end tuberculosis” outlines strategies for promoting meaningful partnerships between health systems and communities and can be more fully used to inform Guideline development [45].

Our study has some limitations. First, classifications do not perfectly capture diversity. Authors may work in institutions outside their countries of origin, possibly resulting in an over- or under-estimate of representation. To avoid overstating underrepresentation of LLMIC/high-MDR-burden country-affiliated individuals, we favored affiliations in those settings in our counts. Additionally, authors and GDG members from HICs often have valuable, relevant clinical and research experience, sometimes in LLMIC/high-burden settings. Using country does not capture other characteristics important to improved representation (rural/urban, religious, ethnic, economic, etc.) in knowledge generation. World Bank income levels are crude distinctions that do not interrogate the structures leading to placement in the categories [46]. And, they mask important intra-category and both inter- and intra-country differences. Second, peer-reviewed article authorship and GDG membership represent small areas within the larger space of knowledge generation and control; investigations of funding sources, data ownership, and membership in other decision-making bodies would augment the present work. Lastly, we acknowledge that this work was initiated in the U.S, a low-TB-burden HIC. In fact, the decolonizing global health movement has been largely driven by academics in HICs [47]. We recognize that the burden of condemning and rectifying injustices often falls upon those with the least power to transform repressive systems. Our intention is, therefore, to leverage our privilege to initiate a dialogue within the MDR/RR-TB academic and activist community and invite perspectives from those within our discipline who have been adversely affected. Engaging in meaningful discourse and listening deeply to our peers is a powerful first step toward change.

## Conclusion

This research highlights the epistemic injustice of the imbalance in opportunity to generate and use knowledge between those who directly bear the brunt of the MDR/RR-TB burden globally and those who do not. Transformative changes proposed in the burgeoning literature on decolonizing global health could promote more equitable representation in groups that define policy. Future research is necessary to evaluate whether such changes produce better-aligned guidance—and accelerate its uptake—for the treatment of MDR/RR-TB.

## Supplementary Information

Supplementary Material 1.

## Data Availability

The datasets used and/or analyzed during the current study are available from the corresponding author on reasonable request.

## Abbreviations

GDG: Guideline Development Group
HIC: High-income country
IPD: Individual patient data
LIC: Low-income country
LLMIC: Low- and lower-middle-income countries
LMIC: Lower-middle-income country
MDR/RR-TB: Multi-drug resistance tuberculosis
NIAID: National Institute of Allergy and Infectious Disease
NIH: National Institutes of Health
NTP: National Tuberculosis Program
OA: Open access
SAP: Structural Adjustment Programs
UMIC: Upper-middle income country
WHO: World Health Organization
