# Supplementary material for: Revealing disparities in representation in knowledge generation and guideline development
Source: BMC Health Serv Res. 2024 Nov 30;24:1516. doi: 10.1186/s12913-024-11958-1 (PMC11607871; doi:10.1186/s12913-024-11958-1)
Supplement: Supplementary file 1 — Supplementary Material 1. [file 12913_2024_11958_MOESM1_ESM.docx]

Table S1. Complete list of GDG members and their classifications between 2016-2022

| **Guideline Year** | **# GDG member from country** | **Country of GDG Member Institutional Affiliation** | **World Bank Classification** | **MDR-TB high burden?** |
| --- | --- | --- | --- | --- |
| 2016 | 1 | Armenia | LMIC | No |
| 2016 | 1 | Belarus | UMIC | Yes |
| 2016 | 1 | Canada | HIC | No |
| 2016 | 1 | Colombia | UMIC | No |
| 2016 | 1 | Dominican Republic | UMIC | No |
| 2016 | 1 | Georgia | LMIC | No |
| 2016 | 1 | Italy | HIC | No |
| 2016 | 1 | Netherlands | HIC | No |
| 2016 | 1 | Pakistan | LMIC | Yes |
| 2016 | 1 | Rwanda | LIC | No |
| 2016 | 1 | Spain | HIC | No |
| 2016 | 1 | UK | HIC | No |
| 2016 | 7 | USA | HIC | No |
| 2016 | 1 | Viet Nam | LMIC | Yes |
| 2018 | 1 | Brazil | UMIC | No |
| 2018 | 2 | Canada | HIC | No |
| 2018 | 1 | Dominican Republic | UMIC | No |
| 2018 | 2 | Eswatini | LMIC | No |
| 2018 | 1 | Ghana | LMIC | No |
| 2018 | 2 | India | LMIC | Yes |
| 2018 | 1 | Italy | HIC | No |
| 2018 | 1 | Mexico | UMIC | No |
| 2018 | 1 | Pakistan | LMIC | Yes |
| 2018 | 1 | Papua New Guinea | LMIC | Yes |
| 2018 | 1 | Peru | UMIC | Yes |
| 2018 | 1 | Philippines | LMIC | Yes |
| 2018 | 1 | Romania | UMIC | No |
| 2018 | 1 | South Africa | UMIC | Yes |
| 2018 | 1 | Thailand | UMIC | Yes |
| 2018 | 1 | UK | HIC | No |
| 2018 | 4 | USA | HIC | No |
| 2020 | 1 | Australia | HIC | No |
| 2020 | 1 | Belarus | UMIC | Yes |
| 2020 | 1 | Brazil | UMIC | No |
| 2020 | 1 | Canada | HIC | No |
| 2020 | 1 | China | UMIC | Yes |
| 2020 | 1 | Dominican Republic | UMIC | No |
| 2020 | 1 | Estonia | HIC | No |
| 2020 | 1 | Eswatini | LMIC | No |
| 2020 | 1 | India | LMIC | Yes |
| 2020 | 1 | Indonesia | LMIC | Yes |
| 2020 | 1 | Iran | LMIC | No |
| 2020 | 1 | Italy | HIC | No |
| 2020 | 1 | Mexico | UMIC | No |
| 2020 | 1 | Netherlands | HIC | No |
| 2020 | 1 | Niger | LIC | No |
| 2020 | 1 | Northern Ireland | HIC | No |
| 2020 | 1 | Pakistan | LMIC | Yes |
| 2020 | 1 | Peru | UMIC | Yes |
| 2020 | 5 | South Africa | UMIC | Yes |
| 2020 | 1 | Tanzania | LMIC | No |
| 2020 | 5 | USA | HIC | No |
| 2020 | 1 | Viet Nam | LMIC | Yes |
| 2022 | 1 | Bangladesh | LMIC | Yes |
| 2022 | 1 | Brazil | UMIC | No |
| 2022 | 1 | Canada | HIC | No |
| 2022 | 1 | China | UMIC | Yes |
| 2022 | 1 | Estonia | HIC | No |
| 2022 | 1 | Eswatini | LMIC | No |
| 2022 | 2 | France | HIC | No |
| 2022 | 1 | Germany | HIC | No |
| 2022 | 1 | India | LMIC | Yes |
| 2022 | 1 | Indonesia | UMIC | Yes |
| 2022 | 1 | Iran | LMIC | No |
| 2022 | 1 | Italy | HIC | No |
| 2022 | 1 | Kenya | LMIC | No |
| 2022 | 1 | Myanmar | LMIC | Yes |
| 2022 | 1 | Pakistan | LMIC | Yes |
| 2022 | 1 | Philippines | LMIC | Yes |
| 2022 | 3 | South Africa | UMIC | Yes |
| 2022 | 2 | UK | HIC | No |
| 2022 | 3 | USA | HIC | No |
| 2022 | 1 | Viet Nam | LMIC | Yes |
